# Impact of Hydrodynamic Injection and phiC31 Integrase on Tumor Latency in a Mouse Model of MYC-Induced Hepatocellular Carcinoma

**DOI:** 10.1371/journal.pone.0011367

**Published:** 2010-06-29

**Authors:** Lauren E. Woodard, Annahita Keravala, W. Edward Jung, Orly L. Wapinski, Qiwei Yang, Dean W. Felsher, Michele P. Calos

**Affiliations:** 1 Department of Genetics, Stanford University School of Medicine, Stanford, California, United States of America; 2 Division of Oncology, Department of Medicine, Stanford University School of Medicine, Stanford, California, United States of America; Saint Louis University, United States of America

## Abstract

**Background:**

Hydrodynamic injection is an effective method for DNA delivery in mouse liver and is being translated to larger animals for possible clinical use. Similarly, ϕC31 integrase has proven effective in mediating long-term gene therapy in mice when delivered by hydrodynamic injection and is being considered for clinical gene therapy applications. However, chromosomal aberrations have been associated with ϕC31 integrase expression in tissue culture, leading to questions about safety.

**Methodology/Principal Findings:**

To study whether hydrodynamic delivery alone, or in conjunction with delivery of ϕC31 integrase for long-term transgene expression, could facilitate tumor formation, we used a transgenic mouse model in which sustained induction of the human *C-MYC* oncogene in the liver was followed by hydrodynamic injection. Without injection, mice had a median tumor latency of 154 days. With hydrodynamic injection of saline alone, the median tumor latency was significantly reduced, to 105 days. The median tumor latency was similar, 106 days, when a luciferase donor plasmid and backbone plasmid without integrase were administered. In contrast, when active or inactive ϕC31 integrase and donor plasmid were supplied to the mouse liver, the median tumor latency was 153 days, similar to mice receiving no injection.

**Conclusions/Significance:**

Our data suggest that ϕC31 integrase does not facilitate tumor formation in this *C-MYC* transgenic mouse model. However, in groups lacking ϕC31 integrase, hydrodynamic injection appeared to contribute to *C-MYC*-induced hepatocellular carcinoma in adult mice. Although it remains to be seen to what extent these findings may be extrapolated to catheter-mediated hydrodynamic delivery in larger species, they suggest that caution should be used during translation of hydrodynamic injection to clinical applications.

## Introduction

Hydrodynamic injection of plasmid DNA involves a rapid, high-volume injection of DNA into the tail vein of mice [1], [2]. This method can provide delivery of DNA to as many as 40% of hepatocytes and has been widely adopted for delivery of nucleic acids to mouse liver. In addition, catheter-mediated adaptations of the method have been developed for DNA delivery in larger animals, opening the possibility of clinical use for gene therapy [3]. Several groups have reported successful gene delivery to the pig liver [4]–[7], and a Phase I clinical trial has been conducted in thrombocytopenia patients [6].

Use of ϕC31 integrase in conjunction with hydrodynamic delivery offers a strategy to make gene delivery in hepatocytes permanent, by bringing about covalent integration of the plasmid DNA into the chromosomes [8], [9]. ϕC31 integrase is a large serine recombinase that is capable of integrating *attB*-containing donor plasmids into pseudo *attP* sites that occur endogenously in mammalian chromosomes [10]. Because its mechanism of integration requires DNA sequence recognition, ϕC31 integrase has a more restricted integration profile than other integrating vectors such as retroviruses and transposons [11]. The more limited number of potential integration sites may make ϕC31 integrase less likely to activate an oncogene or disrupt a tumor suppressor gene.

Both hydrodynamic injection and ϕC31 integrase are relatively new technologies that have not yet been rigorously tested for their potential tumorigenicity. To date, hydrodynamic delivery has not been associated with increased cancer risk. Similarly, ϕC31 integrase has been used in many pre-clinical gene therapy studies over the years, involving hundreds of animals, without evidence of cancer incidence [10]. In a recent study, ϕC31-modified human cord-lining epithelial cells failed to form tumors in SCID mice [12]. The same study also analyzed microarray data and found that three tumor suppressor gene transcripts were upregulated in ϕC31-modified cells [12]. Nevertheless, after prolonged expression of ϕC31 integrase in cultured cells, chromosomal rearrangements were found by both plasmid rescue and karyotyping [11], [13]–[15]. If such aberrations occurred *in vivo*, they could increase cancer risk by contributing to genomic instability. Therefore, it was of interest to analyze with greater sensitivity whether exposure to hydrodynamic injection or ϕC31 integrase could stimulate tumorigenesis in an appropriate animal model.

In studies not designed to evaluate cancer risk, cancers have appeared after injection of viral gene therapy vectors *in utero* or in neonatal mice [16], [17]. By contrast, in this study we specifically tested whether hydrodynamic injection and/or ϕC31 integrase were capable of contributing to MYC-induced tumorigenesis in adult mice in a previously validated animal model. This approach is similar to studies that have investigated the potential contribution of various gene therapy vectors to blood cancer formation, which occurred during a clinical trial that employed retroviral vectors to treat children with X-linked severe combined immunodeficiency [18]–[20]. Small molecule carcinogens, shRNA, and partial hepatectomy have all been demonstrated in separate studies to contribute to MYC-induced hepatocellular carcinoma using the same model and similar methods to those used in this study [21]–[23].

Mice transgenic for both *TRE-MYC* [24] and *LAP-tTA* [25] have been developed as a mouse model for hepatocellular carcinoma in which the human C-MYC transcription factor is expressed in the liver when doxycycline is absent [26]. In this mouse model, the tumor latency is long enough that subtle oncogenic perturbations would be detectable, yet short enough to be experimentally tractable [23]. *C-MYC* is genomically amplified in up to 50% of human liver tumors, and this amplification can result in C-MYC overexpression [27], [28]. The C-MYC transcription factor plays a key role in development by inducing genes that control cell division, growth, and apoptosis [29]. When disregulated in blood cancers, C-MYC has been shown to contribute to the formation of double-strand breaks [30]. Hepatocellular carcinomas initiated in this model were found to regress when C-MYC expression was terminated [26], [31]. We asked whether hydrodynamic delivery, either with or without ϕC31 integrase, might cooperate with C-MYC to accelerate tumor formation in this mouse model.

## Materials and Methods

### Ethics Statement

The Stanford Administrative Panel on Laboratory Animal Care approved all procedures performed on animals in protocol number 9477, assurance number A3213-01. The Stanford Comparative Medicine program is accredited by the Association for Accreditation and Assessment of Laboratory Animal Care International.

### Plasmids

pCS, pCSmI, and pCSI have been described previously [32], [33]. Briefly, these plasmids carry ampicillin resistance and contain a CMV promoter and SV40 poly-A tail (pCS), between which either mutant S20F (pCSmI) or wild-type (pCSI) ϕC31 integrase was cloned. pLiLucB is a liver-specific, luciferase-expressing *attB* donor plasmid that was constructed by digesting pNBL2 [34] with *Xho*I to remove the CMV promoter and digesting pVFB [35] with *EcoR*I to obtain the human alpha-1 antitrypsin promoter with an apolipoprotein enhancer. The ends of both fragments were made blunt by filling in with Klenow polymerase, and the pNBL2 backbone was treated with phosphatase. The construct was ligated using T4 ligase and checked by *Xcm*I digestion and sequencing of the promoter region.

### Mouse experiments

Genotyped *LAP-tTA* homozygous females were bred to genotyped *TRE-MYC* males (the *TRE-MYC* transgene is on the Y chromosome) to give male mice having one copy of each gene. All mice were on the FVB/N strain background. A solution of doxycycline hyclate (Sigma-Aldrich, St. Louis, MO) was given as drinking water at a concentration of 100 µg/ml from one week before mating cages were set up until after weaning at 7–8 weeks of age. Autoclaved paper tubes were placed in mouse cages to prevent fighting. Mice were randomly assigned to groups in random order. Each experimental group consisted of mice from between 3–8 separate litters. Hydrodynamic injections were carried out as previously described [36] using a heat lamp for tail vein dilation, with the exception that sterile phosphate-buffered saline #20012 (Invitrogen, Carlsbad, CA) was used to dilute the DNA. Live animal imaging was done on the IVIS 200 machine (Caliper Life Sciences, Alameda, CA). Animals in groups given luciferase were imaged on one of eight separate imaging schedules, consisting of between 1–3 experimental groups per imaging schedule. The mice were imaged at Day 1, Week 1, 2, 4, 6, 8, 10, 12, 14, 17, 20, etc. until sacrifice. Mice were monitored weekly for abdominal swelling and/or other signs of distress and sacrificed when it was believed that they would not survive another week. After dissection, portions of the normal liver, liver tumors, and metastatic tumors (if present) were fixed for histology as previously described [36], and snap frozen in a dry ice/isopropanol bath for subsequent genomic DNA and protein analysis.

### Luciferase assay

Protein was prepared from tissue samples using the lysis buffer previously described [35] and homogenized with a Kontes pellet pestle motor (VWR, Batavia, IL). Protein prepared from transfected HeLa cells (ATCC, Manassas, VA), producing luciferase from the pNBL2 plasmid after FuGene 6 (Roche, Indianapolis, IN) transfection, was used as the positive control. Protein concentrations were determined by the Bradford method with Protein Assay Reagent (Biorad, Hercules, CA). 15 µg of protein were added per well. The luciferase assay kit procedure was run in quadruplicate on a 96-well plate reader according to the manufacturer's instructions (Promega, Madison, WI).

### PCR analysis

Total DNA was prepared from normal-appearing liver and tumor samples using the DNeasy Blood and Tissue Kit according to the manufacturer's directions, including the optional addition of RNase (Qiagen, Valencia, CA). DNA concentrations were measured using the Nanodrop (Thermo Scientific, Wilmington, DE). 200 ng of DNA was added to each PCR reaction, prepared according to manufacturer's directions using illustra puReTaq Ready-To-Go PCR beads (GE Healthcare, Piscataway, NJ). To detect the luciferase-bearing plasmid pLiLucB in the genomic DNA, the forward primer 5′GACCGTGACCTACATCGTC and the reverse primer 5′-CATGTCTGCTCGAAGCGGC were used to amplify the luciferase gene. The template used for the second round in the nested mpsL1 PCR was 1 µl of a 1∶10 dilution of the first round PCR reaction. Primers to detect integration at mpsL1 and to detect GAPDH have been previously described [37]. The reactions were carried out at least twice for all samples.

## Results

In our previous studies, dozens of wild-type mice have received hydrodynamic injections, with or without integrase [8], [35], [36], [38]. Of these mice, as well as many more mice treated similarly in lab studies that have not been published, no liver tumors have ever been observed. Therefore, for greater sensitivity, a mouse model was used in which all mice would develop tumors. Different groups were tracked to provide survival times that could be compared statistically to determine if the treatments had an effect on the length of time between induction of *C-MYC* expression in the liver and sacrifice due to tumor burden, a time frame defined in this study as “tumor latency.” We hypothesized that since ϕC31 integrase is associated with chromosomal aberrations in tissue culture, its expression might decrease tumor latency in these tumor-prone mice.

**Figure 1:**
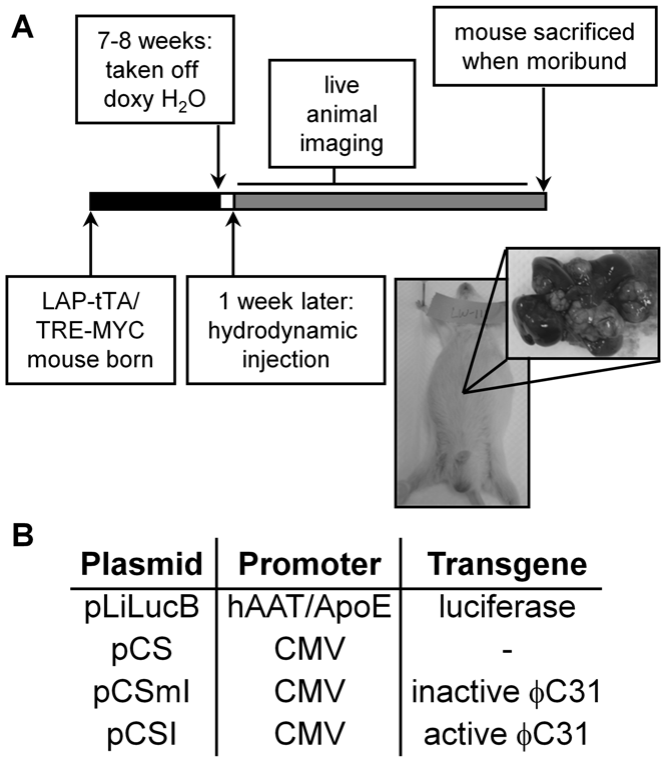
Experimental design of tumorigenesis assay. (**a**) Transgenic mice were taken off of doxycycline drinking water at 7–8 weeks of age to induce expression of the human *C-MYC* transgene specifically in the liver from the LAP promoter, except for one control group (MYC off). Exactly one week after C-MYC induction, all groups except one control group (MYC on, no injection) were given hydrodynamic injections of phosphate-buffered saline alone or DNA plasmids diluted in phosphate-buffered saline. Mice were monitored weekly, imaged every two or three weeks, and sacrificed when tumors were detectable by gross distention of the abdomen as pictured. Inset shows the dissected liver and tumors from the pictured mouse, which was representative of all mice in all groups. (**b**) The plasmids given by hydrodynamic injection and their features.

*LAP-tTA/TRE-MYC* double transgenic, male mice were given doxycycline drinking water from conception until 7–8 weeks of age to suppress C-MYC expression in the liver during development, which would have been lethal (**Fig. 1a**) [22]. Complete regulation of C-MYC expression by doxycycline was previously observed to have a time scale of four days [22], [26]. Therefore, at one week after initiation of sustained C-MYC induction, a hydrodynamic injection [1], [2] was administered containing no plasmid (saline only) or 20 µg each of a luciferase-expressing donor plasmid (pLiLucB) and plasmids containing vector backbone alone (pCS), expressing inactive integrase (pCSmI), or active wild-type integrase (pCSI) (**Fig. 1b**). One group was given the active integrase plasmid pCSI alone (20 µg). The DNA dose of 20 µg per plasmid has been used to confer therapeutic levels of hFIX using ϕC31 integrase in mice [9]. Two groups were not given hydrodynamic injection: one was given doxycycline drinking water for 7–8 weeks, and another was given doxycycline drinking water for one year. Mice that were injected with pLiLucB were imaged the next day to confirm high levels of luciferase expression in the liver, indicating a successful hydrodynamic injection. C-MYC induction preceded integrase expression because the integrase protein can only be detected by Western blot for up to one day after hydrodynamic injection [39].

Mice were monitored weekly for tumor formation. The animals were sacrificed when it was expected that they would not have survived another week, as indicated by swelling in the upper abdomen (**Fig. 1a**) or signs of morbidity. Most of the mice with extensive hepatocellular carcinoma appeared behaviorally normal until the point of sacrifice. At autopsy, mice were dissected, photographed, and examined for the presence of liver tumors. Most tumors were multifocal, presumably arising from different tumor-forming cells (**Fig. 1a**), as has been suggested previously [22], [23], [26]. No differences in gross type, number, size, mass or distribution of tumors were observed between groups. Imaging was done every 2–3 weeks to monitor whether luciferase expression was observed in locations outside of the liver, indicating a possible luciferase-positive metastasis. Although several cases of metastasis were observed upon dissection (**Table 1**), none were detectable by luciferase imaging.

**Table 1:** Treatment group and number of days from initiation of C-MYC overexpression until sacrifice (survival time) for each case of metastasis.

Group	Number of metastasis	Survival time (days)
no injection	1	209
saline injection	0	
pCS/pLiLucB	0	
pCSmI/pLiLucB	3	111, 139, 329
pCSI/pLiLucB	1	258
pCSI alone	1	164

**Figure 2:**
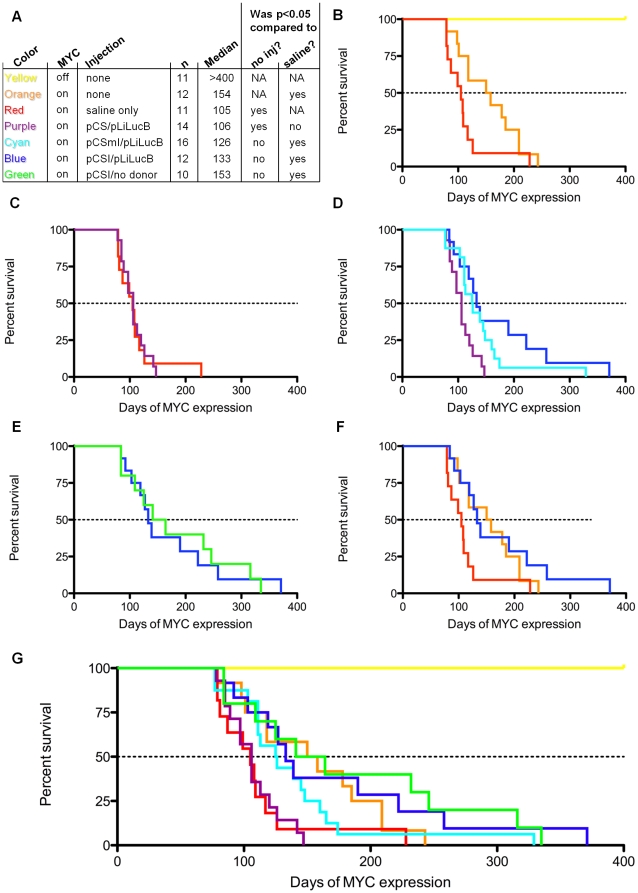
Survival curves suggest that hydrodynamic injection may contribute to C-MYC-induced tumor formation in the mouse liver. (**a**) Key showing the number of animals (n), median survival time in days (Median), and statistical results for each group. The significance as determined by the Gehan-Breslow-Wilcoxon test comparing each group to the MYC on, no injection (no inj?) or MYC on, saline injection (saline?) control groups is given. (**b**) A comparison of MYC off, no injection (yellow), MYC on, no injection (orange), and MYC on, saline injection (red) survival curves. (**c**) A comparison of MYC on, saline injection and MYC on, pCS/pLiLucB (purple) injection survival curves. (**d**) A comparison of MYC on, pCS/pLiLucB, MYC on, pCSmI/pLiLucB (cyan), and MYC on, pCSI/pLiLucB (blue) survival curves. (**e**) The survival curves of groups given pCSI with and without (green) donor plasmid. (**f**) A comparison of pCSI/pLiLucB to the control groups of no injection and saline-only injection. (**g**) The survival curves of all groups shown on the same plot. All plots and statistics were done using GraphPad Prism software.

The seven groups of mice were observed for tumor formation to distinguish the effects of C-MYC induction, hydrodynamic injection, DNA administration, ϕC31 integrase protein, and ϕC31 integrase activity (**Fig. 2a**). To make these effects easier to evaluate, we have separated the composite Kaplan-Meier survival curve (**Fig. 2g**) into several plots comparing these effects in a step-wise manner.

### Hydrodynamic delivery significantly decreased tumor latency

The survival times of mice in the control groups are compared in Figure 2b. The animals that did not receive C-MYC induction or any injections (yellow) survived until the end of the study, 400 days after 7–8 weeks of age. In contrast, induction of C-MYC expression in the liver beginning at adulthood (7–8 weeks) resulted in all of the mice being sacrificed prior to the end of the study with a median tumor latency of 154 days. These results confirmed that the *LAP-tTA/TRE-MYC* mouse model allowed for tight oncogene regulation.

Interestingly, in the group of mice that received a hydrodynamic injection of phosphate-buffered saline without any DNA present, the median tumor latency was only 105 days. There was one mouse that survived past 200 days that may have been an exception in some way, for example, by receiving an unsuccessful hydrodynamic injection. Similarly, a single long-surviving mouse was seen in other groups. To keep these outliers from having major effects on statistical significance, we chose the Gehan-Breslow-Wilcoxon statistical test to compare survival times, because this test gives less weight to later events. The group in which *C-MYC* was turned “on” and no treatment was given (orange) was statistically different than the group that had *C-MYC* “on” and received saline-only hydrodynamic injection (red; p = 0.0359), indicating that there was a significant decrease in tumor latency associated with the hydrodynamic delivery method.

### DNA delivery, luciferase expression, and imaging did not affect tumor latency

To test the effect on tumor latency of DNA without the integrase gene, we gave the transgenic mice hydrodynamic injections containing 20 µg each of pCS and pLiLucB (**Fig. 1b**). Inclusion of this group was intended to control for both the integrase plasmid backbone elements as well as firefly luciferase expression and imaging every 2–3 weeks, which entailed injections of luciferin and anesthesia with isoflurane for a period of approximately 15 minutes. As shown in Figure 2c, the saline-only (red) and pCS/pLiLucB (purple) groups had nearly identical survival curves, except for one late survivor in the saline-only group. The pCS/pLiLucB group was statistically significantly different than the MYC “on”, no injection group (p = 0.014), again implicating effects of the hydrodynamic injection.

### Integrase expression resulted in similar tumor latency to that of untreated mice

To evaluate the effect of ϕC31 integrase protein expression independent of integration activity, we injected the plasmids pCSmI and pLiLucB (**Fig. 1b**) into a group of C-MYC expressing mice (**Fig. 2d**, cyan). The pCSmI plasmid has a S20F mutation in the catalytic serine, rendering the ϕC31 integrase made by this plasmid unable to recombine DNA. We observed a statistically significant increase in tumor latency when inactive integrase protein was present, compared to the saline-only group (p = 0.045). Again, note that we observed a very late surviving mouse in this group, which lived about twice as long as the second-longest surviving mouse in the group.

When the pCSI construct encoding active integrase (**Fig. 1b**) was administered with the pLiLucB donor plasmid (blue, **Fig. 2d**), the tumor latency also increased compared to the saline only group. According to the Gehan-Breslow-Wilcoxon test of statistical significance, the pCSmI/pLiLucB and pCSI/pLiLucB groups were not significantly different. To test if recombination of plasmid DNA was necessary for the observed survival benefit, we also administered 20 µg of pCSI without any *attB* donor plasmid (green) to a cohort of *TRE-MYC/LAP-tTA* mice. Presence of the *attB*-containing plasmid appeared to have no effect on survival (**Fig. 2e**).

To summarize, the *C-MYC* “on”, pCSI/pLiLucB group was not statistically different than the *C-MYC* “on”, no injection group (**Fig. 2f**), indicating that the presence of integrase appeared to counteract the tumor acceleration due to hydrodynamic injection. Hydrodynamic injection without integrase expression yielded a survival curve that was significantly different than the uninjected and pCSI/pLiLucB groups. The acceleration of tumor formation in mice that received a hydrodynamic injection appeared to be somehow abrogated by expression of the integrase. All groups are graphed together in **Fig. 2g** on a longer x-axis.

### Tumors did not have luciferase activity or ϕC31 integrase-mediated integration events

**Figure 3:**
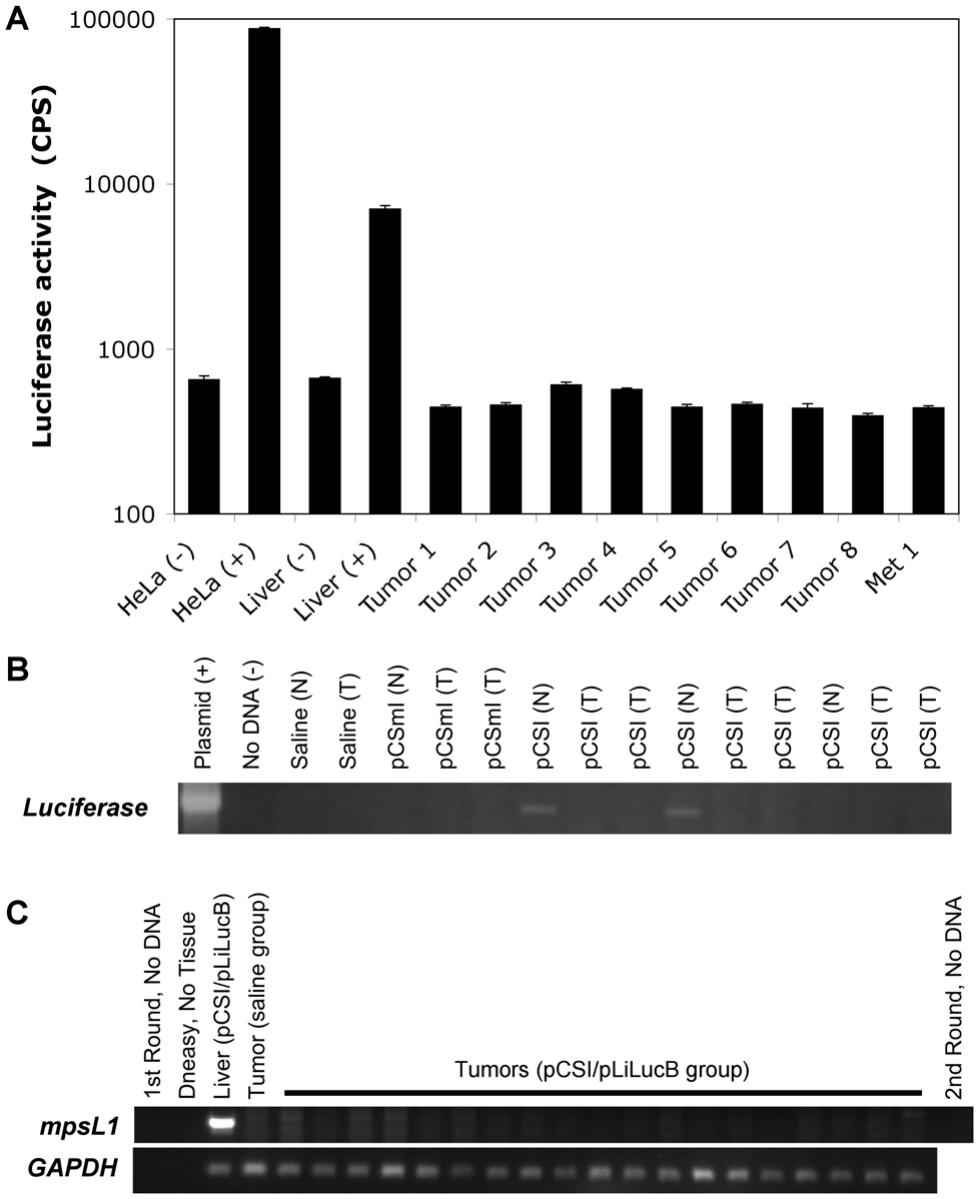
Luciferase activity and PCR analysis of tumors from mice in the pCSI/pLiLucB group provide no evidence of ϕC31 integrase activity. (**a**) Protein extracts were prepared and the luciferase activity was measured in absolute counts per second (CPS). Controls included HeLa cells given FuGene 6 alone [HeLa (-)] or the CMV-luciferase plasmid pNBL2 via FuGene 6 [HeLa (+)], the normal-appearing part of the tumor-ridden liver taken from either a saline-injected mouse [Liver (-)] and pCSI/pLiLucB-injected mouse [Liver (+)]. Eight tumor samples (Tumor 1 through 8) and one metastasis (Met 1) that were obtained from four animals were also analyzed. The error bars give standard error of the mean for four replicates of each sample. (**b**) PCR analysis to detect the pLiLucB plasmid by amplification of the luciferase transgene. Plasmid DNA (20 ng pLiLucB) and no DNA controls show specific amplification of luciferase only in the reaction containing plasmid. One mouse each from the saline-only and pCSmI/pLiLucB groups was analyzed for transgene presence in normal-appearing (N) and tumor (T) tissues (none found). Three mice in the pCSI/pLiLucB group were analyzed for transgene presence in normal-appearing (N) and tumor (T) tissues. Luciferase could be detected in 2/3 normal-appearing liver samples and none of the tumors. (**c**) PCR analysis for integration at the mpsL1 pseudo *attP* site was done on 18 tumors (lanes 5 through 22) and one metastasis (lane 23) taken from nine mice given pCSI/pLiLucB by hydrodynamic injection. Controls included no DNA (1^st^ round, lane 1 and 2^nd^ round, lane 25), and a DNeasy performed on no tissue (lane 2) to show no contamination from the DNA isolation procedure. Normal-appearing liver from a mouse in the pCSI/pLiLucB group (lane 3) served as the positive control. DNA isolated from a tumor in the saline-only group served as the negative control (lane 4). PCR for the *GAPDH* gene showed that sufficient DNA was added to all reactions. Seven tumors and one metastasis were subjected to the analysis in *both* **a** and **c**. Six tumors were analyzed by all assays (**a**, **b** and **c**).

In order to investigate further whether ϕC31 integrase played any role in tumor formation, we analyzed tumors isolated from mice in the pCSI/pLiLucB group. We tested protein extracts from eight tumors and one metastasis from four mice in this group and found that none of them were positive for luciferase activity (**Fig. 3a**). Additionally, we were unable to detect the luciferase gene by PCR in the six tumors from three mice in the pCSI/pLiLucB group that were tested (**Fig. 3b**). This PCR would detect integration at any location in the genome. The tumors that tested negative for luciferase activity included the six tumors that tested negative for the presence of the luciferase gene, suggesting that the luciferase donor plasmid was not integrated and silenced in these tumors. To determine further if ϕC31 integrase mediated integration of plasmid DNA into the cells that generated tumors, we tested all dissected tumors for integration into the dominant pseudo *attP* site in the mouse liver genome known as mpsL1 [9], using a nested PCR. For each round, one primer was in the mouse genome at mpsL1 while the other primer was the in *attB* sequence in the donor plasmid. PCR analysis was performed on DNA isolated from 19 tumors from 9 mice, including 7 tumors and 1 metastasis that were also tested for luciferase activity and six tumors also tested by PCR for the presence of the luciferase gene. No mpsL1 integration was detected in any tumors. Integration was detected only in DNA isolated from the normal-appearing part of the liver (**Fig. 3c**). A GAPDH control demonstrated that a sufficient amount (200 ng) of genomic DNA was added to each PCR reaction. Thus, no evidence for ϕC31 integrase activity was found in any of the tumors from mice in the pCSI/pLiLucB group, suggesting that ϕC31 integrase may not have played a role in tumor formation in the *LAP-tTA/TRE-MYC* transgenic mouse model.

### Luciferase imaging data and correlation to tumor latency

**Figure 4:**
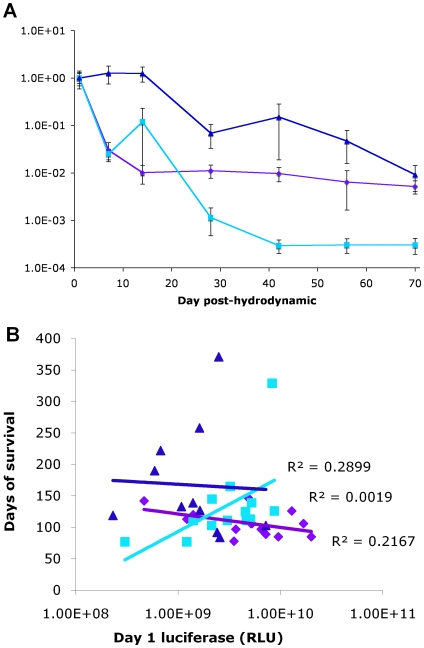
Luciferase expression and the relationship between initial expression values and long-term survival. (**a**) *TRE-MYC/LAP-tTA* transgenic mice given pCS/pLiLucB (purple diamonds), pCSmI/pLiLucB (cyan squares), or pCSI/pLiLucB (blue triangles) by hydrodynamic injection were imaged at Day 1, Week 2, 4, 6, 8, and 10. The normalized luciferase levels were obtained by dividing the average luciferase expression in reflective light units (RLU) at each time point by the average level at day 1 for that group. Propagation of errors was used to determine the standard error at each time point given the division calculation (error bars). By student's t-test of the values at day 70, the pCSI/pLiLucB group and pCS/pLiLucB group had a p-value of 0.067, while the pCSI/pLiLucB group and pCS/pLiLucB group were significantly higher than the pCSmI/pLiLucB group (p = 0.014 and p = 0.000031, respectively). (**b**) Each mouse is represented by one point on the scatterplot, using the day 1 luciferase value in reflective light units (RLU) as the x-coordinate and the days of survival as the y-coordinate. The symbols and colors are identical to those used in **a**. The linear line-of-best-fit was calculated by GraphPad Prism and is plotted for each group (R-squared values of pCS/pLiLucB, 0.1065; pCSmI/pLiLucB, 0.3839; pCSI/pLiLucB, 0.0033). No R-squared values exceeded 0.95, which would have indicated that there was a trend relating transfection efficiency and survival.

Mice were imaged on day 1 and every two weeks thereafter for luciferase expression for the first 14 weeks, followed by every three weeks thereafter. We have shown the luciferase imaging data to week 10 (**Fig. 4a**), because after week 10 (day 70) animals began to be sacrificed, thus complicating the data with increasing statistical error as the group sizes decreased. Averaged luciferase values were normalized to the day 1 luciferase value to remove variability on account of transfection efficiency. The standard error was calculated using propagation of errors to take this normalization into account. pCSI/pLiLucB gave significantly higher long-term expression than pCSmI/pLiLucB (Student's t-test, p = 0.014), demonstrating that ϕC31 integrase was active in the mouse liver. The pCS/pLiLucB and pCSmI/pLiLucB groups would still retain some luciferase expression due to random integration of the pLiLucB plasmid. The pCS/pLiLucB group maintained luciferase values that were significantly higher than pCSmI/pLiLucB (Student's t-test, p = 3×10^−5^). It is unclear why these groups have different long-term luciferase levels. One could speculate that since the pCS/pLiLucB group developed tumors faster than the groups given integrase-expressing plasmids, increased numbers of luciferase-positive cells in the liver cause higher long-term luciferase levels. We do not believe that luciferase is an ideal readout for overall levels of transgene expression in the liver, because the levels detected are dependent on the distance from the surface of the animal. In this animal model, tumors formed that may have complicated interpretation of the luciferase levels by displacing the normal liver away from the surface of the animal. It was the possibility of finding metastases that motivated our use of the luciferase transgene in the liver. However, luciferase imaging did not detect any luciferase-positive metastases (**Table 1**).

In order to correlate the efficiency of hydrodynamic injection with tumor latency, the luciferase expression on day 1 was plotted against survival time (**Fig. 4b**). An R-squared value exceeding 0.95 would have indicated a trend correlating transfection efficiency and survival. The R-squared values for all groups were lower than 0.95, regardless of the method used to calculate the trend line, suggesting that variations in transfection efficiency may not have affected tumor latency.

## Discussion

This study represents a novel use of a genetic mouse model to provide insights into the safety of new gene therapy methodologies in a solid tissue. Such mouse models are available for most organs that may be targeted by different gene therapy methods. Similar studies using tumor-prone mouse models to investigate the incidence of blood cancers after treatment with various viral vectors have been used to demonstrate that newer vectors containing insulator elements, weaker promoters, and/or lentiviral sequences may be safer than those vectors originally used in clinical trials for X-SCID that became implicated in the formation of leukemias [18], [19].

Our data suggested an acceleration in tumor formation due to hydrodynamic injection in combination with C-MYC overexpression. The mechanism whereby hydrodynamic injection stimulated tumor formation is unknown. However, one could speculate that the extensive cellular proliferation that occurred after hydrodynamic injection in wild-type mice [36] may play a role. A comparable level of cellular proliferation induced by partial hepatectomy was reported to cause a similar acceleration in tumor formation in this cancer model [22], [23].

We hypothesized that if ϕC31 integrase were tumorigenic, one would have expected a further decrease in tumor latency when hydrodynamic injection was accompanied by ϕC31 integrase. Instead, no measurable increase in C-MYC-induced tumor formation was found, suggesting that our hypothesis that ϕC31 integrase would be tumorigenic in this animal model was incorrect. No tumors taken from mice given active ϕC31 integrase and the luciferase donor plasmid were found to have integrated at a preferred pseudo *attP* site in the mouse genome, even though one or two out of nineteen might have been expected to be positive by random chance. It is unknown why the presence of either active or inactive ϕC31 integrase reduced the tumorigenicity of hydrodynamic injection. The recombination activity of ϕC31 integrase was not likely to be responsible for the significantly longer tumor latency compared to the pCS/pLiLucB and saline groups, because the pCSmI/pLiLucB, pCSI/pLiLucB, and pCSI alone groups were not statistically different from one another. Because the presence of ϕC31 integrase protein in hepatocytes was correlated with reduced cellular proliferation in a previous study [36], regardless of integrase activity, one possible hypothesis is that the decreased levels of proliferation resulted in reduced tumorigenesis in groups receiving integrase plasmids. We could also speculate that the immune system played a role in the effect, or that interaction of ϕC31 integrase with cellular proteins [40], [41] proved to be anti-tumorigenic. It could be suggested that hydrodynamic injection did not transfect the cells that can go on to become cancer. However, when two oncogenes (*MET* and *ΔN90-CTNNB1*) were administered to the liver of wild-type mice via hydrodynamic injection and integrated with a transposon system, tumors developed in most of the mice within 200 days [42]. Thus, hydrodynamic injection has already been shown to be capable of delivering oncogenes to tumor-forming cells within the liver.

Our mouse model revealed a statistically significant contribution of the hydrodynamic method itself, with or without DNA, to tumor formation. Although significant, it should be noted that the decrease in tumor latency from hydrodynamic delivery alone (**Fig. 2b**) was modest compared to the dramatic one-week median tumor latency when shRNA or small molecule carcinogens such as carbon tetrachloride cooperated with C-MYC in this mouse model [23]. While hydrodynamic injection is perhaps the most robust method of plasmid DNA delivery to mouse liver currently available, adaptations of the method that are less disruptive would be desirable for clinical use. For example, localized, catheter-mediated delivery to the liver has been explored in large animal models [3] and may have a superior safety profile compared to the systemic delivery method used in mice.
